# Expression of transmembrane protein 26 (TMEM26) in breast cancer and its association with drug response

**DOI:** 10.18632/oncotarget.9493

**Published:** 2016-05-20

**Authors:** Norbert Nass, Angela Dittmer, Vicky Hellwig, Theresia Lange, Johanna Mirjam Beyer, Benjamin Leyh, Atanas Ignatov, Christine Weiβenborn, Tove Kirkegaard, Anne E. Lykkesfeldt, Thomas Kalinski, Jürgen Dittmer

**Affiliations:** ^1^ Otto-von-Guericke-Universität Magdeburg, Institut für Pathologie, Magdeburg, Germany; ^2^ Klinik für Gynäkologie, Martin-Luther-Universität Halle-Wittenberg, Halle/Saale, Germany; ^3^ Otto-von-Guericke-Universität Magdeburg, Universitätsfrauenklinik, Magdeburg, Germany; ^4^ Breast Cancer Group, Cell Death and Metabolism, Danish Cancer Society Research Center, Copenhagen, Denmark; ^5^ Present address: Department of Surgery, Koege Hospital, Koege, Denmark; ^6^ Breast Cancer Group, Cell Death and Metabolism, Danish Cancer Society Research Center, Copenhagen, Denmark

**Keywords:** estrogen receptor, anti-estrogen resistance, sonic hedgehog, integrin beta1, triple-negative breast cancer

## Abstract

We have previously shown that stromal cells desensitize breast cancer cells to the anti-estrogen fulvestrant and, along with it, downregulate the expression of TMEM26 (transmembrane protein 26). In an effort to study the function and regulation of TMEM26 in breast cancer cells, we found that breast cancer cells express non-glycosylated and N-glycosylated isoforms of the TMEM26 protein and demonstrate that N-glycosylation is important for its retention at the plasma membrane. Fulvestrant induced significant changes in expression and in the N-glycosylation status of TMEM26. In primary breast cancer, TMEM26 protein expression was higher in ERα (estrogen receptor α)/PR (progesterone receptor)-positive cancers. These data suggest that ERα is a major regulator of TMEM26. Significant changes in TMEM26 expression and N-glycosylation were also found, when MCF-7 and T47D cells acquired fulvestrant resistance. Furthermore, patients who received aromatase inhibitor treatment tend to have a higher risk of recurrence when tumoral TMEM26 protein expression is low. In addition, TMEM26 negatively regulates the expression of integrin β1, an important factor involved in endocrine resistance. Data obtained by spheroid formation assays confirmed that TMEM26 and integrin β1 can have opposite effects in breast cancer cells. These data are consistent with the hypothesis that, in ERα-positive breast cancer, TMEM26 may function as a tumor suppressor by impeding the acquisition of endocrine resistance. In contrast, in ERα-negative breast cancer, particularly triple-negative cancer, high TMEM26 expression was found to be associated with a higher risk of recurrence. This implies that TMEM26 has different functions in ERα-positive and -negative breast cancer.

## INTRODUCTION

Breast cancer, the most common diagnosed cancer in women around the world [1], is a heterogeneous disease which can be subtyped by immunochemical or molecular analysis [2]. Immunohistochemically, the ERα-positive tumor subtype, mostly also positive for PR, is distinguished from the Her-2 (human epidermal receptor-2)-positive and the triple-negative tumor (negative for all three receptors). To treat patients with ERα-positive tumors, endocrine therapy is commonly applied by either using anti-estrogens or aromatase inhibitors [3]. Her-2-positive tumors are targeted by Her-2 directed antibodies or kinase inhibitors [4]. Resistance to these drugs is a major obstacle in the success of these treatments [5, 6].

Drug resistance can be achieved by many mechanisms [6–8]. They can be triggered by tumor-residing stromal cells as the result of their interaction with tumor cells [9]. For instance, carcinoma-associated fibroblasts (CAFs) or mesenchymal stem (stromal) cells (MSCs) can cause ERα-positive breast cancer cells to resist the growth inhibitory effect of anti-estrogens by causing these cells to activate the PI3K (phosphoinositol-3-kinase)/AKT-signaling pathway by inducing downregulation of the IGF (insulin-like growth factor)-regulating protein IGFBP5 (IGF binding protein 5) [10]. The partial loss of IGFBP5 may give rise to additional, IGF-independent effects, such as an increase in Bcl-3 (B-cell leukemia/lymphoma 3) expression that can also contribute to anti-estrogen resistance [10].

Along with the decline in the IGFBP5 level, the expression of other genes was found to be changed [10]. One of these genes was *tmem26* (transmembrane protein 26) [10], a gene present in the genomes of human and mouse as well as in *Drosophila* [11]. Its product is a membrane protein predicted to contain five to eight transmembrane domains. Though expressed during murine embryogenesis, it does not seem to be essential for embryo survival. In adult mice, the TMEM26 protein has been identified as a surface marker for the so-called beige (brite) fat cell, which is distinct from the classical white and brown adipocytes [12]. The functions of TMEM26 are still unknown. TMEM26 is also expressed in cancer. In pancreatic carcinoma, higher TMEM26 RNA levels were shown to correlate with poorer outcome [13].

Here, we studied TMEM26 RNA and protein expression in breast cancer cell lines, examined TMEM26 protein expression in breast cancer samples and analyzed its potential importance for endocrine resistance. Our data suggest that TMEM26 is an N-glycosylated protein whose expression and N-glycosylation status is regulated by ERα. As a negative regulator of integrin β1, TMEM26 may suppress the development of endocrine resistance.

## RESULTS

### TMEM26 is expressed in ERα-positive and -negative breast cancer cell lines

**Figure 1:**
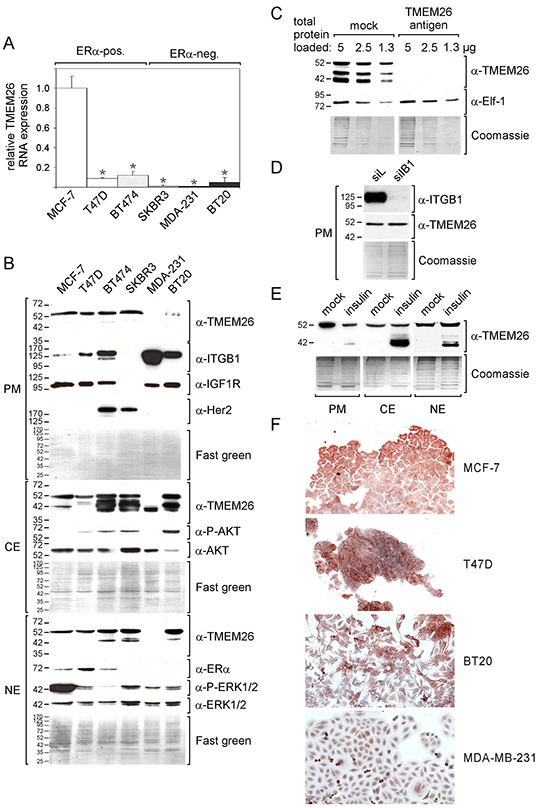
TMEM26 RNA and protein are expressed in ERα-positive and -negative breast cancer cell lines **A. B.** ERα-positive (pos.) and -negative (neg.) breast cancer cell lines were examined for TMEM26 RNA expression by Q-RT-PCR (A) and for TMEM26 protein expression by Western blot analysis after proteins had been fractionated (PM = plasma membrane fraction, CE = cytosolic fraction and NE = nuclear fraction) (B). (A) Statistical analyses of Q-PCR data were performed by student's *t*-test (*p < 0.05). Each bar represents the mean value ± S.D. of at least three independent experiments. (B) TMEM26 protein expression patterns were compared with the expression of various proteins and phospho-proteins (ITGB1 = integrin β1, IGF1R = insulin-like growth factor receptor 1, Her2 = human epidermal receptor 2, AKT, P-AKT = phospho-AKT, ERα = estrogen receptor a, ERK1/2 and P-ERK1/2 = phospho-ERK1/2). The blots were stained with Fast green to check for equal protein loading. **C.** The specificities of the interactions between the anti-TMEM26 antibody and the three major proteins p40^TMEM26^, p44^TMEM26^ and p53^TMEM26^ were analyzed by the preincubating the anti-TMEM26 antibody with TMEM26 antigen in a molar ratio of ~1:50. For control reasons, the effect of the TMEM26 antigen on the interaction of the anti-Elf-1 antibody with the Elf-1 protein was also studied. **D.** Following transfection of MCF-7 cells with either the integrin β1-specific siRNA siIB1 or the control siRNA siL, the plasma membrane fraction was analyzed for TMEM26 and integrin β1 expression by Western blot analysis. **E.** MCF-7 cells were treated with insulin or mock for three days and analyzed for TMEM26 protein expression by Western blot analysis after protein fractionation. (C-E) To check for equal protein loading, proteins remaining in the gel after blotting were stained with Coomassie Blue. **F.** Immunocytochemical analyses of adherent MCF-7, T47D, BT20 and MDA-MB-231 cells for the expression of TMEM26.

The finding that desensitization of ERα-positive breast cancer cells to the anti-estrogen fulvestrant was accompanied by a decline in TMEM26 RNA expression [10] prompted us to compare TMEM26 expression in ERα-dependent and ERα-independent breast cancer cell lines. Measurements of the TMEM26 RNA levels in three ERα-positive (MCF-7, T47D and BT474) and three ERα-negative breast cancer cell lines (SKBR3, MDA-MB-231 and BT20) revealed that TMEM26 RNA levels are significantly higher in the ERα-positive breast cancer cell lines (Figure 1A). The highest level was found in MCF-7 cells, the lowest level in MDA-MB-231 cells. The ERα/Her2 status of the different cell lines was confirmed by Western blot analysis (Figure 1B).

To measure TMEM26 protein levels in these cell lines, we carried out Western blot analyses by using an anti-TMEM26 antibody that recognizes the C-terminal part of the TMEM26 protein. The TMEM26 protein is predicted to contain a number of membrane domains (http://www.ch.embnet.org/software/TMPRED_form.html) (Supplementary Figure S1) and may therefore preferentially be located in the plasma membrane. Hence, we performed the analyses with three separate subcellular protein fractions, a plasma membrane, cytosolic and nuclear fraction. In five of the six cell lines, a ~53 kD anti-TMEM26-reactive protein (from now on called p53^TMEM26^) could be visualized in all three protein fractions (Figure 1B). In addition, a number of faster migrating anti-TMEM26-reactive proteins, most prominently a ~40 kD and a ~44 kD protein (from now on called p40^TMEM26^ and p44^TMEM26^, respectively), could be detected in the cytosolic and nuclear fractions. Though no obvious association between the expression of these proteins and the ERα status could be observed, it was striking that, in contrast to the other cell lines tested, the two triple-negative cell lines BT20 and MDA-MB-231 showed only barely detectable levels of p53^TMEM26^ in the plasma membrane. In addition, the two Her2-expressing cell lines BT474 and SKBR3 and the triple-negative cell line BT20 cells expressed p40^TMEM26^ and/or p44^TMEM26^ in the cytosol at much higher levels than ERα-positive MCF-7 and T47D cells. Furthermore, of all cell lines tested, the MDA-MB-231 cell line was the only one that exclusively expressed p40^TMEM26^.

To confirm that the anti-TMEM26 antibody specifically recognized TMEM26 protein, we compared anti-TMEM26 reactivity in the presence and absence of the same peptide (PrEST antigen TMEM26) that was used to generate the antibody. Once the anti-TMEM26 antibody had been preincubated with this TMEM26 antigen, it was unable to detect p40^TMEM26^, p44^TMEM26^ and p53^TMEM26^ (Figure 1C). To show that this blocking effect of the TMEM26 antigen on the anti-TMEM26 antibody was specific, we reprobed the blot with an anti-Elf-1 (Ets-like factor 1) antibody that recognizes the transcription factor Elf-1 [14]. Clearly, the TMEM26 antigen was unable to interfere with the interaction between the anti-Elf-1 antibody and the 80 kD Elf-1 protein. These data demonstrate that p40^TMEM26^, p44^TMEM26^ and p53^TMEM26^ are indeed TMEM26 protein isoforms.

We next sought to analyze whether the differences in TMEM26 protein expression patterns as observed between breast cancer cell lines may be associated with differences in the activities of certain signaling pathways or with differences in the expression of specific proteins. In this analysis, we focused on the phospho-proteins P-AKT and P-ERK1/2, which indicate the activities of the PI3K/AKT and Ras/Raf/MEK/ERK1/2 pathways, respectively, and on the proteins integrin β1 and IGF1R (IGF1 receptor). These proteins were chosen, because their levels have been shown to be altered along with TMEM26 expression when MCF-7 cells were desensitized to fulvestrant by stromal cells [10]. The analyses revealed that the two triple-negative cell lines BT20 and MDA-MB-231, which express little or no plasma membrane p53^TMEM26^, show the highest level of integrin β1. To explore the possibility that high integrin β1 expression may lead to low plasma membrane levels of p53^TMEM26^, we analyzed the effect of an integrin β1-specific siRNA (siIB1), which fully abrogated integrin β1 expression (Figure 1D), on p53^TMEM26^ expression in the plasma membrane of MCF-7 cells. There was no difference in p53^TMEM26^ expression between cells transfected with siIB1 and cells transfected with the control siRNA siL (Figure 1D) suggesting that integrin β1 does not regulate p53^TMEM26^ expression. Another finding was that the cell lines BT474, SKBR3 and BT20, which express the highest levels of p40^TMEM26^ and/or p44^TMEM26^ of all cell lines tested, show also the highest levels of P-AKT (Figure 1B). To test whether the PI3K/AKT pathway is able to regulate TMEM26 protein expression, we incubated MCF-7 cells with insulin for three days, a treatment shown to activate the PI3K/AKT pathway in these cells while leaving the P-ERK1/2 levels and the TMEM26 RNA expression unaffected [10]. Insulin caused the appearance of faster migrating TMEM26 proteins, predominantly of p40^TMEM26^, in all protein fractions (Figure 1E). In the plasma membrane fraction, the insulin-induced appearance of p40^TMEM26^ was accompanied by a decline in the p53^TMEM26^ level. These data suggest that the PI3K/AKT pathway regulates TMEM26 protein expression post-transcriptionally by promoting the expression of faster migrating TMEM26 protein isoforms.

We next analyzed TMEM26 protein expression *in situ* by performing immunocytochemical analysis of two ERα-positive cell lines (MCF-7, T47D) and two ERα-negative cell lines (BT20, MDA-MB-231). By using the same anti-TMEM26 antibody as used for Western blot analysis, TMEM26-specific immunoreactivity could be detected in the cytoplasm of MCF-7, T47D and BT20 cells (Figure 1F). Though BT20 cells express much more cytosolic p44^TMEM26^ than MCF-7 and T47D cells (Figure 1B), the TMEM26-specific staining intensities obtained by immuncytochemistry was similar between these cell lines. This may suggest that, in immunocytochemistry, the anti-TMEM26 antibody recognizes predominantly p53^TMEM26^. This assumption is supported by the finding that MDA-MB-231 cells, which express considerable levels of cytosolic p40^TMEM26^, but are deficient of p53^TMEM26^ (and also p44^TMEM26^), showed little TMEM26-specific immunoreactivity (Figure 1F). Within the cytoplasm, TMEM26-specific immunoreactivity was either located close to the plasma membrane (MCF-7, T47D) or close to the nucleus in the perinuclear area (BT20). Interestingly, the nucleus itself was free of TMEM26, although TMEM26 protein could be detected in the nuclear protein fraction by Western blot analysis. This discrepancy may be explained by the likelyhood that the nuclear fraction also contained proteins of the nuclear membrane.

### TMEM26 is an N-glycosylated protein in breast cancer cells

**Figure 2:**
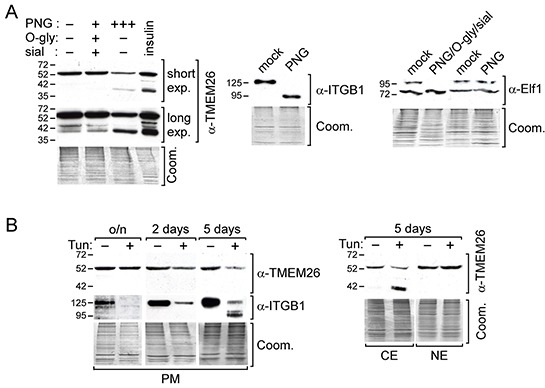
p44^TMEM26^ and p53^TMEM26^ are N-glycosylated TMEM26 proteins **A.** Proteins isolated from MCF-7 cells were either incubated with peptid-N-glycosidase F (PNG) alone or in combination with O-glycosidase (O-gly) and sialidase (sial) or mock-treated at 37°C o/n and analyzed for TMEM26, integrin β1 (ITGB1) or Elf-1 protein expression patterns by the Western blot technique. For comparison reasons, along with these samples, proteins isolated from insulin-treated MCF-7 cells were also analyzed for TMEM26 expression. **B.** Following treatment of MCF-7 cells with tunicamycin (Tun, 5μg/ml) for o/n, 2 or 5 days, TMEM26 protein expression pattern was determined in the plasma membrane (PM) (left panel), cytosolic (CE) (right panel) and nuclear fraction (NE) (right panel) by Western blot analysis. **A, B.** To check for equal protein loading, proteins remaining in the gel after blotting were stained with Coomassie Blue (Coom.). Exp. = exposure.

We next searched for the reason(s) that give rise to the different TMEM26 protein isoforms. TMEM26 has been reported to belong to a group of proteins that are N-glycosylated in Jurkat T-cells [15]. The glycosylation site has been determined to be amino acid N-110. Together with the amino acids Q-111 and T-112, it forms a classical recognition site (N-X-S/T) for the addition of N-glycans [16]. This motif is located in a domain that most likely extrudes from the cell membrane (Supplementary Figure S1). To test if TMEM26 is also N-glycosylated in breast cancer cells, we treated MCF-7 cell protein extracts with N-glycosylase (PNGase F) alone or with a mixture of PNGase F, O-glycosylase and sialidase. Clearly, PNGase F alone substantially reduced the level of p53^TMEM26^ and of p44^TMEM26^ and, in the same time, increased the level of p40^TMEM26^ (Figure 2A). For comparison, we also tested the effects of these enzymes on the plasma membrane-residing N-glycosylated 125 kD integrin β1 protein [17] and on the 98 kD O-glycosylated transcription factor Elf-1 located in the nucleus together with its non-glycosylated 80kD form [14]. PNGase F induced a complete shift from the 125 kD N-glycosylated integrin β1 form to the non-glycosylated 95 kD isoform, while it had no effect on the level of O-glycosylated Elf-1 protein (Figure 2A). However, treatment of O-glycosylase and sialidase removed the 98 kD Elf-1 protein from the nuclear fraction. Collectively, these data show that TMEM26 is also N-glycosylated in breast cancer cells and further suggest that p53^TMEM26^ and p44^TMEM26^ are N-glycosylated derivatives of p40^TMEM26^.

N-glycosylation is important for the retention of glycoproteins at the cell surface [18]. This may explain why the glycosylated p53^TMEM26^ was the predominant form found in the plasma membrane protein fractions (Figure 1B). To test the hypothesis, we incubated MCF-7 cells with the N-glycosylation inhibitor tunicamycin and monitored changes in TMEM26 protein abundance and pattern over a peroid of five days. For comparison, we also analyzed the protein status of integrin β1. Five days of incubation with tunicamycin resulted in a substantial decline in the plasma membrane level of p53^TMEM26^, while two-day-incubation led to a slight decrease and overnight incubation had no effect on the p53^TMEM26^ level (Figure 2B). In contrast, overnight treatment with tunicamycin was sufficient to completely remove the N-glycosylated 125 kD isoform of integrin β1 from the plasma membrane. These data support the notion that N-glycosylation of TMEM26 is indeed important for its retention at the plasma membrane, but also suggest that, compared to integrin β1, TMEM26 is retained much longer at the cell surface. To confirm that tunicamycin blocks N-glycosylation of TMEM26 we also analyzed TMEM26 protein expression in the cytosolic and nuclear fractions. Clearly, five days of incubation with tunicamycin led to a reduced level of p53^TMEM26^ in the cytosolic fraction while, in the same time, giving rise to the appearance of p40^TMEM26^ (Figure 2B). Interestingly, tunicamycin did not affect the p53^TMEM26^ level in the nuclear fraction, suggesting that p53^TMEM26^ is stably integrated in the environment from which the “nuclear fraction proteins” are extracted.

### TMEM26 expression is altered in fulvestrant-resistant cell lines

**Figure 3:**
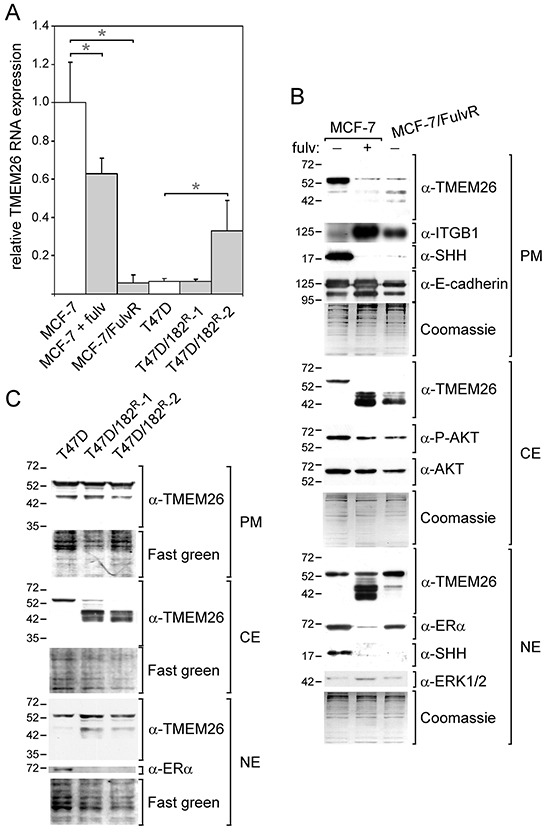
TMEM26 expression is altered in fulvestrant-treated and -resistant ERα-positive breast cancer cell lines TMEM26 RNA and protein expression in fulvestrant (fulv)-treated MCF-7 cells and in fulvestrant-resistant breast cancer cell lines MCF-7/FulvR, T47D/182^R^-1 and T47D/182^R^-2 (grown in the absence of fulvestrant) were compared with TMEM26 expression in the corresponding parental cell line by Q-RT-PCR **A.** or Western blot analysis **B, C.**. (A) Statistical analyses of Q-PCR data were performed by student's *t*-test (* p < 0.05). Each bar represents the mean value ± S.D. of at least three independent experiments. (B, C) The expression status of a number of other proteins and phospho-proteins were also analyzed (E-cadherin, ITGB1 = integrin β1, SHH = sonic hedgehog, AKT, P-AKT = phospho-AKT, ERα = estrogen receptor α, ERK1/2 and P-ERK1/2 = phospho-ERK1/2). **B, C.** To check for equal protein loading, either the proteins that remained in the gel after protein transfer were stained by Coomassie Blue **B.** or proteins transferred to the membranes were stained by Fast Green **C.** (PM = plasma membrane fraction, CE = cytosolic fraction and NE = nuclear fraction).

The finding that stromal cell-induced fulvestrant resistance was accompanied by changes in TMEM26 expression [10] prompted us to study TMEM26 expression in fulvestrant-treated MCF-7 cells and in fulvestrant-resistant MCF-7 and T47D breast cancer cell lines. Though fulvestrant only moderately suppressed TMEM26 RNA expression in MCF-7 cells (Figure 3A), it caused a strong decrease in p53^TMEM26^ abundance in the plasma membrane and cytosolic fractions (Figure 3B). This was accompanied by a strong upregulation of the integrin β1 protein level. Fulvestrant also dramatically increased the levels of p40^TMEM26^ and p44^TMEM26^ in the cytosolic as well as in the nuclear fraction, while leaving the level of p53^TMEM26^ in the nuclear fraction unaffected. In contrast to insulin, which strongly increased the level of P-AKT, fulvestrant decreased the P-AKT level. This could mean that fulvestrant acts on TMEM26 protein expression in a different way than insulin. We wondered whether the SHH (sonic hedgehog)/Gli (glioma-associated oncogene) pathway may be involved in the fulvestrant-induced changes in TMEM26 protein expression for two reasons. First, ERα interacts with SHH in MCF-7 cells [19] and regulates its expression in gastric and endothelial cells [20, 21]. Second, Gli3 regulates TMEM26 expression in the developing murine limb [22]. We found that treatment of MCF-7 cells with fulvestrant resulted in a complete loss of the ~19 kD N-terminal domain of SHH in the plasma membrane and nuclear fractions (Figure 3B). In the cytosolic fraction, SHH was barely detectable (data not shown). Since the SHH/Gli pathway has been reported to cross-talk with the PI3K/AKT pathway [23], we wondered if insulin also affects SHH expression. As shown in Supplementary Figure S2A, insulin reduced the SHH level in the plasma membrane fraction and caused its elimination from the nuclear fraction. Hence, fulvestrant and insulin have in common that they both induce the upregulation of faster migrating TMEM26 protein isoforms while having little or no effect on TMEM26 RNA expression and, in the same time, trigger the loss of (nuclear) SHH. This prompted us to study the role of SHH in the fulvestrant- and insulin-mediated changes in TMEM26 protein expression by using an SHH-specific siRNA (siSHH), which strongly reduced plasma membrane and nuclear SHH protein levels (Supplementary Figure S2B). In siSHH-treated MCF-7 cells, the level of cytosolic p53^TMEM26^ was lower than in cells transfected with control siRNA (siL) (Supplementary Figure S2B). Concomitantly, siSHH increased the levels of p40^TMEM26^ in the cytosolic and nuclear fractions. Though these effects were reminiscent of those induced by fulvestrant and/or insulin, they were by far not as strong. Complicating the interpretation of these results, siSHH decreased the level of ERα (Supplementary Figure S2B), which means that it cannot be ruled out that siSHH affected TMEM26 protein expression indirectly by downregulating the ERα level. Similar to fulvestrant, siSHH also reduced the level of P-AKT and increased that of integrin β1. Again, it remains unclear whether the decline in the ERα level or the loss of SHH was responsible for these changes. Different to fulvestrant and insulin, siSHH failed to induce an upregulation of the level of p44^TMEM26^ and increased the plasma membrane level of p53^TMEM26^. In summary, the positive feedback loop between ERα and SHH does not allow a conclusion as to whether SHH mediates some of the effects of ERα on TMEM26 protein expression.

For the analysis of TMEM26 protein expression in fulvestrant-resistant breast cancer cell lines, cells were grown in fulvestrant-free medium for two weeks before proteins were extracted to avoid direct influence of fulvestrant on TMEM26 expression. Basically, the TMEM26 protein expression patterns in the fulvestrant-resistant cell line MCF-7/FulvR resembled those in fulvestrant-treated MCF-7 parental cells, except that the levels of the faster migrating TMEM26 proteins were much lower in the nuclear fraction from MCF-7/FulvR cells (Figure 3B). Also like fulvestrant-treated MCF-7 cells, MCF-7/FulvR cells show high expression of integrin β1 and no expression of SHH. However, different to fulvestrant-treated MCF-7 cells, MCF-7/FulvR cells express TMEM26 RNA at a much lower level than parental untreated MCF-7 cells (Figure 3A). This low expression of TMEM26 RNA may have contributed to the altered TMEM26 protein expression pattern in MCF-7/FulvR cells.

T47D-derived fulvestrant-resistant cell lines T47D/182^R^-1 and T47D/182^R^-2 share with the MCF-7/FulvR cell line the high expression of p40^TMEM26^ and p44^TMEM26^ in the cytosolic and/or nuclear fractions and the absence of cytosolic p53^TMEM26^ (Figure 3C). However, different to MCF-7/FulvR cells, T47D/182^R^-1 and T47D/182^R^-2 cells express p53^TMEM26^ in the plasma membrane at similar levels as the parental cells (Figure 3C), do not show lower levels of TMEM26 RNA than the parental cells (Figure 3A) and are completely deficient of ERα (Figure 3C). Of note, the SHH protein is not detectable in T47D cells, neither in the parental, nor in the fulvestrant-resistant cells (data not shown), excluding the possibility that SHH is involved in the changes in TMEM26 protein expression associated with fulvestrant resistance in T47D cells. Collectively, these data show that fulvestrant resistance is accompanied by distinct changes in TMEM26 protein expression. All three fulvestrant-resistant cell lines tested have in common that, in the cytosol, p53^TMEM26^ is replaced by p40^TMEM26^ and p44^TMEM26^. It is possible that the loss of ERα activity is the reason for the altered TMEM26 protein expression in the fulvestrant-resistant T47D and MCF-7 cell lines, since T47D/182^R^-1 and T47D/182^R^-2 cells do not express ERα and since MCF-7/FulvR cells are deficient of SHH, which likely indicates that the ERα expressed in these cells is not active.

### Downregulation of TMEM26 RNA expression changes TMEM26 protein expression pattern

**Figure 4:**
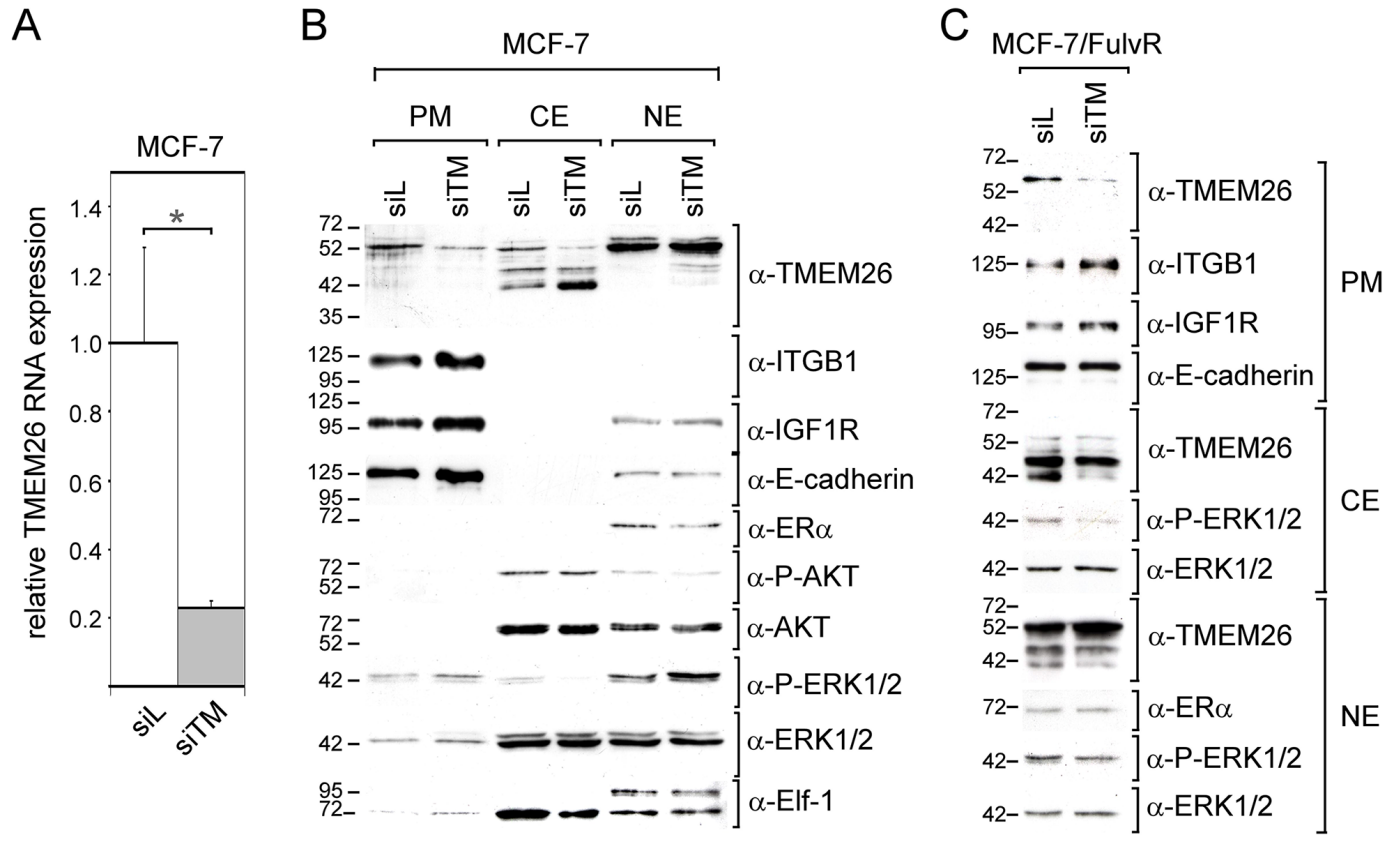
Knock-down of TMEM26 leads to changes in TMEM26 protein expression and to an increase in the integrin β1 level **A.** The effect of the TMEM26-specific siRNA siTM on the TMEM26 RNA expression in MCF-7 cells as measured by Q-RT-PCR, siL = control siRNA. **B, C.** Western blot analyses of the expression of certain proteins and phospho-proteins (TMEM26, ITGB1 = integrin β1, IGF1R = insulin-like growth factor receptor 1, E-cadherin, AKT, P-AKT = phospho-AKT, ERα = estrogen receptor α, ERK1/2 and P-ERK1/2 = phospho-ERK1/2, Elf-1 = Ets-like factor-1) in the plasma membrane (PM), cytosolic (CE) and nuclear fractions (NE) as prepared from siTM- or siL-transfected MCF-7 (B) or MCF-7/FulvR cells (C).

Though it is likely that loss of ERα activity has caused the changes in TMEM26 protein expression in MCF-7/FulvR cells, it cannot be ruled out that the very low TMEM26 RNA level in these cells has also contributed to these changes. To investigate this possibility, we transfected MCF-7 cells with an TMEM26-specific siRNA (siTM), which decreased TMEM26 RNA level by ~5-fold (Figure 4A). Along with its effect on TMEM26 RNA expression, siTM decreased the p53^TMEM26^ level in the plasma membrane and cytosolic fractions, but did not affect the p53^TMEM26^ level in the nuclear fraction (Figure 4B). In addition, siTM caused an increase in the abundance of cytosolic p40^TMEM26^ and induced the appearance of p44^TMEM26^ in the nuclear fraction. Hence, the TMEM26 protein expression pattern in siTM-transfected MCF-7 cells is very similar to that in MCF-7/FulvR cells (Figure 3B). This suggests that a strong reduction in TMEM26 RNA level is sufficient to cause a TMEM26 protein expression pattern as is found in MCF-7/FulvR cells.

It was of interest to study whether siTM would still have an effect on TMEM26 protein expression pattern, once the TMEM26 RNA level is as low as in MCF-7/FulvR cells. Indeed, siTM reduced the plasma membrane p53^TMEM26^ level also in MCF-7/FulvR cells, and, in addition, eliminated p40^TMEM26^ from the cytosolic and nuclear fractions, while the level of p44^TMEM26^ remained unaffected (Figure 4C). Hence, even at low basal TMEM26 RNA level, changes in the TMEM26 RNA level are translated into changes in TMEM26 protein expression.

We next explored the possibility that siTM also affects the expression of the proteins and phospho-proteins that we have studied before (Figure 1B). Interestingly, siTM upregulated the plasma membrane abundance of integrin β1 and IGF1R in both MCF-7 cells and MCF-7/FulvR cells (Figure 4B, 4C). Furthermore, siTM induced a decline in cytoplasmic levels of P-ERK1/2 in MCF-7 and MCF-7/FulvR cells, which was accompanied by an increase in nuclear P-ERK1/2 levels in MCF-7 cells (Figure 4B, 4C). To confirm that cytosolic and nuclear proteins were well separated from each other by the fractionation method we have used, we analyzed the expression of Elf-1. In accordance to previous data [24], the larger O-glycosylated 98 kD isoform was only found in the nucleus, while the non-glycosylated 80 kD form was present in both nucleus and cytoplasm. Interestingly, siTM caused the nuclear level of ERα in MCF-7 cells to decrease, while it had no effect on ERα expression in MCF-7/FulvR cells. This differential effect by siTM on ERα in the two cell lines may explain why siTM increased cytosolic p40^TMEM26^ expression in MCF-7 cells while decreasing it in MCF-7/FulvR cells. In MCF-7 cells, the direct downregulating effect of siTM on the p40^TMEM26^ level may have been compromised by the indirect upregulating effect of siTM as induced by the decline in the ERα level. It is unlikely that the PI3K/AKT signaling pathway was involved in this indirect effect of siTM, since the P-AKT level was not changed by siTM in MCF-7 cells (Figure 4B).

Collectively, these data indicate that a change in the TMEM26 RNA level has a strong effect on TMEM26 protein expression. However, it may affect the levels of the different TMEM26 isoforms differently, probably depending on whether the downregulation of TMEM26 RNA is accompanied by a change in ERα expression.

### Downregulation of TMEM26 expression increases integrin β1 expression and delays spheroid formation

**Figure 5:**
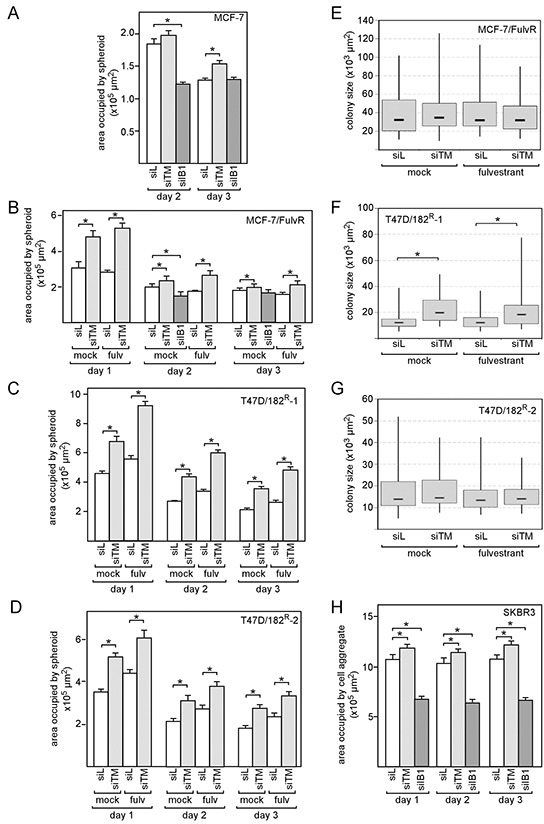
Knock-down of TMEM26 results in a delay of spheroid formation by breast cancer cells A-D, H MCF-7 (A), MCF-7/FulvR (B), T47D/182^R^-1 (C), T47D/182^R^-2 (D) and SKBR3 cells (H) were analyzed for their abilities to aggregate in 3D cultures in the presence of the TMEM26-specific siRNA siTM, the integrin β1-specific siRNA siIB1 or the control siRNA siL and in the presence or absence of fulvestrant. As a measure for the size of the spheroid the area occupied by the spheroid was determined as described in Materials and methods. Each bar represents the mean value ± S.D. of at least three independent experiments. **E-G.** Colony assays were performed for MCF-7/FulvR (E), T47D/182^R^-1 (F) and T47D/182^R^-2 cells (G) to assess whether siTM affects cell growth. For each condition, the sizes of at least 50 single colonies were determined. Statistical analyses were performed by student's *t*-test (A-D, H) or Wilcoxon matched pair test (E-G), * p < 0.05. ITGB1 = integrin β1, IGF1R = insulin-like growth factor 1 receptor.

The negative effect of siTM on integrin β1 expression may suggest that TMEM26 modulates integrin β1 function. To explore this possibility, we performed spheroid formation assays. This assay was chosen because integrin β1 is known to be involved in spheroid formation [25]. In this assay, cells were first transfected with siTM, siIB1 or control siRNA (siL), kept under adherent culture conditions for two days and then transferred to 3D culture conditions to allow the cells to form a spheroid, a process which usually takes three days. The sizes of the developing spheroid were monitored daily for a total of three days. In the presence of siTM, spheroids formed by MCF-7 cells were significantly larger on day 3 than those formed by control (siL)-treated MCF-7 cells, whereas siIB1-transfected cells aggregated much faster, completing spheroid formation already after two days (Figure 5A). Similar data were obtained when the experiments were repeated with MCF-7/FulvR cells (Figure 5B). siTM also delayed cell aggregation of the two fulvestrant-resistant T47D cells (Figure 5C, 5D). Since it could not be ruled out that, during cell aggregation, also cell proliferation took place, which may then have affected the size of the aggregate, we tested whether TMEM26 modulates cell proliferation. To address this issue, we used a colony growth assay, in which adherent cells were allowed to form individual clones and to expand over a period of six days. No difference in colony size could be observed between siTM- and siL-transfected MCF-7/FulvR and T47D/182^R^-2 cells, irrespective of fulvestrant being present or not (Figure 5E, 5F). In contrast, colony growth of T47D/182^R^-1 was increased by siTM (Figure 5G). This suggest that, except for T47D/182^R^-2 cells, it is unlikely that proliferation played a role in spheroid formation by MCF-7/FulvR and T47D/182^R^-2 cells and that, therefore, spheroid size was most probably a measure of cell aggregation activity. In view of the above results, it was of interest to also examine the effect of TMEM26 and integrin β1 knock-down on aggregation of ERα-negative cells. By using SKBR3 cells, we could also show for ERα-negative cells that siTM increases while siIB1 decreases the average size of cell aggregates (Figure 5H). Of note, after initial compaction during the first day in 3D culture, SKBR3 cells did not further aggregate to form a spheroid. Collectively, these data demonstrate that TMEM26 and integrin β1 have opposing effect on 3D cell aggregation by ERα-positive and -negative breast cancer cells. Since downregulation of TMEM26 increased integrin β1 expression it is likely that TMEM26 regulates spheroid formation through its inhibitory effect on integrin β1.

### ERα-/PR-positive breast cancers show higher TMEM26 protein expression

**Figure 6:**
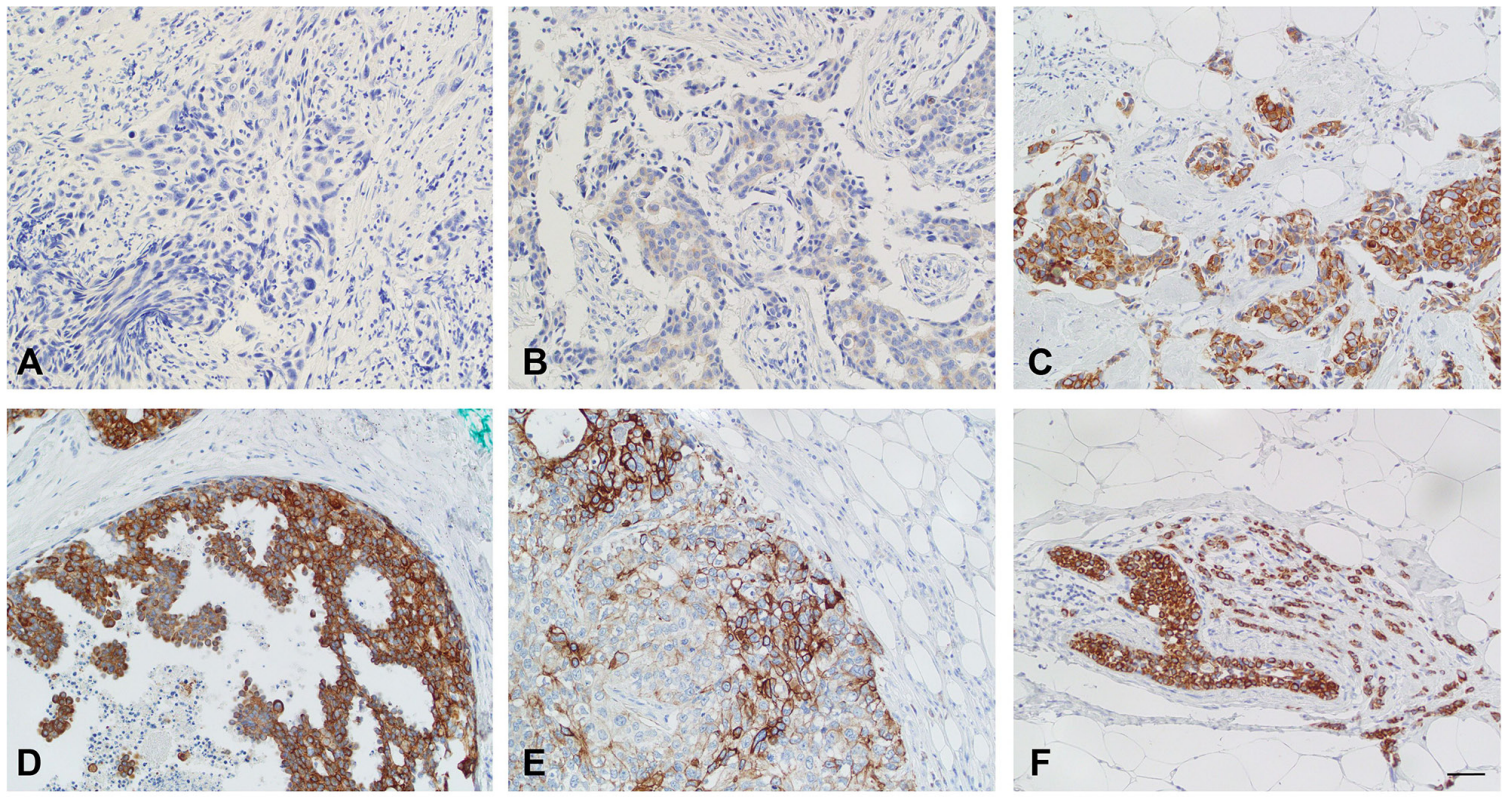
Breast cancer samples show strong differences in TMEM26-specific immunoreactivity TMEM26 specific immunoreactivity of tumor cells in breast cancer samples were determined by immunohistochemistry. **A.** Negative immunostaining (staining intensity = 0), invasive carcinoma of no special type, **B.** weak cytosolic immunostaining (staining intensity = 1) in an invasive carcinoma of no special type, **C.** strong immunostaining (staining intensity = 3), predominantly cytosolic, in an invasive carcinoma of no special type, **D.** strong immunostaining (staining intensity = 3), predominantly cytosolic, in a ductal carcinoma *in situ*; **E.** strong immunostaining (staining intensity = 3), exclusively membranous, in an invasive carcinoma of no special type, **F.** strong immunostaining (staining intensity = 3), predominantly cytosolic, in an invasive lobular carcinoma and lobular carcinoma *in situ*. Bar = 50 μm.

We next examined the TMEM26 protein status in 207 breast cancer specimens by immunohistochemistry. As was found by immunocytochemical analysis of breast cancer cell lines (Figure 1F), TMEM26 specific-immunoreactivity was found in the cytoplasm, but not in the nucleus of the tumor cells (Figure 6). In almost all cases, staining was equally distributed throughout the cytoplasm. Only in a few cases, staining was close to the plasma membrane (an example is shown in Figure 6E). To confirm the specificity of the anti-TMEM26 reactivity in immunohistochemical staining, consecutive sections of a breast cancer specimen were either incubated with TMEM26 antigen-blocked or mock-treated anti-TMEM26 antibody or were not exposed to the anti-TMEM26 antibody. Blocking anti-TMEM26 by the TMEM26 antigen or omitting the incubation step with the anti-TMEM26 antibody prevented TMEM26-specific immunoreactivity (Supplementary Figure S3B, S3C), while mock-treatment had no effect on the activity of the anti-TMEM26 antibody (Supplementary Figure S3D) suggesting that the antibody recognized specifically TMEM26 proteins.

For quantitation of the TMEM26-specific immunoreactivity, staining intensity and area were combined as described in Materials and methods. A score of eight or higher was considered as high expression. Based on this calculation, high anti-TMEM26 immunoreactivity significantly correlated with positivity for ERα and PR (Table 1). Also, TMEM26 expression was significantly higher in post-menopausal as compared to pre-menopausal breast cancer patients. No other correlation of TMEM26-specific immunoreactivity with clinico-pathological factors was observed.

**Table 1:** Comparison of TMEM26 protein expression with clinico-pathological data

Variable	No. of cases	Cases of high TMEM26 expression (%)	p-value^a^
*menopausal status*			
pre-menopausal	46	15 (32.6)	0.007
post-menopausal	161	89 (55.3)	
*Nodal status*			
N0	133	71 (53.3)	0.383
N1	71	33 (46.5)	
*Tumor size*			
pT1	103	55 (53.4)	0.403
pT2-4	103	49 (47.6)	
*Grading*			
G1	20	12 (60.0)	0.13
G2	116	63 (54.3)	
G3	71	30 (40.8)	
*ER*α			
negative	54	16 (29.6)	<0.0001
positive	151	88 (58.3)	
*PR*			
negative	97	39 (40.2)	0.007
positive	110	65 (59.1)	
*Her2*			
negative	154	75 (48.7)	0.44
positive	51	28 (54.9)	

a values were calculated by cross table analysis using the χ^2^ test.

These results imply that TMEM26 protein expression is particularly high in ERα-driven breast cancers.

### TMEM26 may predict success of treatment with aromatase inhibitors

**Figure 7:**
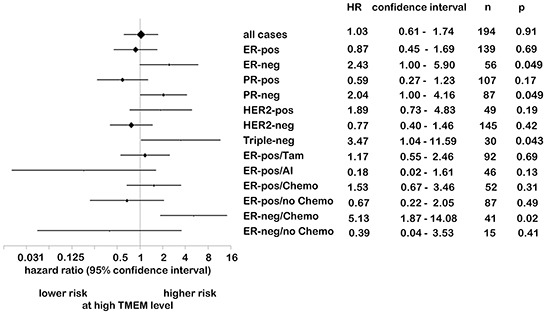
While TMEM26-specific immunoreactivity is not associated with risk of recurrence in ERα-positive cancer, high TMEM26 expression correlates with high risk of recurrence in ERα-negative tumors Forest plot of risk of recurrence stratified by subgroups. The diamonds represent the point estimates of the hazard ratio (HR). The vertical bars show the estimated 95% confidence intervals. The size of the diamond is proportional to the precision of the estimate. ER = estrogen receptor α, PR = progesterone receptor, Her2 = human epidermal receptor 2, TAM, AI, chemo = treatment with tamoxifen, aromatase inhibitor or chemotherapeutics, respectively.

To assess a possible association of anti-TMEM26 immunoreactivity with the risk of recurrence, hazard ratios were calculated and summarized in a Forest plot stratified by hormone receptor status, Her-2 status, and type of adjuvant therapy (Figure 7). For this calculation, the data of 194 of the total of 207 patients were used. No differences in the risk of recurrence were found when the analysis included all 194 patients or when it was limited to the 139 patients who were diagnosed with ERα-positive tumors. When the latter group was stratified by the type of endocrine treatment, again no association between TMEM26 expression and risk of recurrence could be observed when the patients received tamoxifen (N = 92). However, for those patients who were treated with an aromatase inhibitor (N = 46), a trend was found suggesting that a higher risk of recurrence is associated with a lower anti-TMEM26 reactivity. For the group of patients diagnosed with an ERα-negative tumor (N = 56), high tumoral TMEM26 expression was found to be significantly associated with a higher risk of recurrence (~2.4-fold, p = 0.049). Within this group, patients diagnosed with a triple-negative tumor (N = 30, p = 0.043), but not with a Her2-positive tumor (N = 49) showed a higher risk for recurrence at high tumoral TMEM26 expression.

We also performed Kaplan-Meier survival analyses which confirmed the trend that lower tumoral TMEM26 expression was unfavorable for patients who received aromatase treatment, which applied to both recurrence-free and overall survival (Supplementary Figure S4E, S4F). In contrast, high TMEM26 expression was significantly associated with lower recurrence-free (p = 0.015) and overall survival (p = 0.036) in the group of patients who developed triple-negative tumors (Supplementary Figure S5C, S5D). Also patients who were diagnosed with an ERα-negative tumor and who received chemotherapy showed a more unfavorable recurrence-free survival (p < 0.0001) at high tumoral TMEM26 expression (Supplementary Figure S6D). Since these groups overlap, it is not clear whether low TMEM26 is predictive for chemotherapy benefit in ERα-negative tumors or whether it is prognostic for better survival in triple-negative cancer. Collectively, these data show that, in ERα-positive breast cancer, the TMEM26 protein is not an indicator for survival, though it may have a potential predictive value for patients treated with an aromatase inhibitor. In contrast, in ERα-negative cancer, specifically in triple-negative cancer, high TMEM26 is associated with a higher risk of recurrence.

## DISCUSSION

The TMEM26 protein has been reported to be an N-glycosylated protein in Jurkat T-cells [15] and to be a membrane protein in beige fat cells [12]. Here, we show that TMEM26 is also an N-glycosylated protein in breast cancer cells and that N-glycosylation is important for its retention at the plasma membrane. Our data suggest that, of the several isoforms as detected by Western blot analysis, p40^TMEM26^ is the non-glycosylated, 368 amino acids long full length form of TMEM26, from which the two other major isoforms p44^TMEM26^ and p53^TMEM26^ are generated by N-glycosylation. We show that p53^TMEM26^ is the predominant form in the plasma membrane. Data obtained by tunicamycin treatment of MCF-7 cells suggest that the retention time of the N-glycosylated p53^TMEM26^ protein at the cell surface is much higher than that of N-glycosylated integrin β1 (Figure 2A). It is possible that the retention time of p53^TMEM26^ at the plasma membrane is increased by cross-linkage to endogenous lectins, as was shown for other glycoproteins [18]. Immunocytochemical and -histochemical analyses revealed that TMEM26 protein is located in the cytoplasm where it may center around the plasma membrane or around the nucleus, while it is not present within the nucleus. The reason why the TMEM26 protein could be detected in the nuclear fraction by Western blot analysis may lie in the likelyhood that the nuclear fraction contains nuclear membrane proteins.

When we compared TMEM26 RNA and protein expression in a number of ERα-positive and -negative breast cancer cell lines, we noticed a disconnect between TMEM26 RNA and protein expression. While ERα-positive breast cancer cell lines showed higher TMEM26 RNA levels than ERα-negative ones, there seemed to be no obvious association of the expression of any of the TMEM26 protein isoforms with the ERα status in Western blot analysis. Nevertheless, ERα inactivation by fulvestrant resulted in a loss of plasma membrane and/or cytoplasmic p53^TMEM26^ in MCF-7 and T47D cells and, concomitantly, led to an increase in the level of p40^TMEM26^ and p44^TMEM26^ suggesting that ERα is also involved in the regulation of TMEM26 protein expression on the post-transcriptional level. The notion that ERα activity is linked to TMEM26 protein expression is supported by data obtained by immunohistochemical analysis of 207 breast cancer specimens showing a statistically highly significant association between the status of ERα and PR and the status of TMEM26. It is possible that the number of breast cancer cell lines we have studied was not high enough to confirm this connection between ERα and TMEM26 protein expression also with cultured cells. Immunohistochemistry does not allow a distinction between the different TMEM26 protein isoforms. However, based on a comparison between the Western blot data and the immunocytochemical results (Fig. 1B vs. 1D) we concluded that, *in situ*, the anti-TMEM26 antibody recognizes predominantly the p53^TMEM26^ isoform. First, MDA-MB-231 cells, which lack p53^TMEM26^ but express p40^TMEM26^ (Figure 1B), did barely show TMEM26-specific immunoreactivity. Second, BT20, which strongly express p44^TMEM26^ along with p53^TMEM26^, did not display higher TMEM26-specific immunoreactivity than MCF-7 or T47D cells, which primarily express p53^TMEM26^. Based on this assumption and given the likelyhood that ERα positively regulates p53^TMEM26^ expression as suggested by the data obtained by fulvestrant (Figure 3B, 3C), it makes sense that TMEM26-specific immunoreactivity in breast cancer specimens correlates with ERα- and PR-positivity. Though ERα likely promotes p53^TMEM26^ expression, it is not a prerequisite for p53^TMEM26^ expression as exemplified by SKBR3 and BT20, two ERα-negative breast cancer cell lines which highly express this protein (Figure 1B). The PI3K/AKT pathway may also contribute to the regulation of TMEM26 protein expression. This pathway may be particularly important for TMEM26 protein expression in ERα-negative breast cancers, such as the triple-negative breast cancers, which show the highest activity of the PI3K/AKT pathway among all breast cancers [26].

The decline in the p53^TMEM26^ level and the concomitant rise in p44^TMEM26^ and p40^TMEM26^ levels as induced by insulin and fulvestrant suggest that these factors interfere with N-glycosylation of the TMEM26 protein. However, as the levels of p44^TMEM26^ and p40^TMEM26^ are disproportionately raised compared to the loss of p53^TMEM26^, insulin and fulvestrant may additionally increase specifically the stability of p44^TMEM26^ and p40^TMEM26^ proteins. Indeed, both ERα and the PI3K/AKT pathway have been shown to interfere with N-glycosylation and protein stability [27–33]. In the uterus, estrogen has been found to promote N-glycosylation of proteins by increasing the synthesis of mannosylphosphoryldolichol synthase (MPDS), an enzyme that is important for glycoprotein assembly [28]. Since deficiency in MPDS leads to the transfer of truncated oligosaccharides onto the nascent proteins [34], blocking ERα activity by fulvestrant may cause incomplete N-glycosylation of TMEM26. As for protein stability, estrogen administered to MCF-7 cells has been shown to upregulate the expression of RNF115 (E3 ubiquitin ligase RING finger protein 115), a protein that induces proteosomal degradation of proteins, such as the cell cycle inhibitor p21 [29, 35]. Since RNF115 is associated with a positive ERα status in breast cancer specimens [36], a general importance of ERα for the regulation of RNF115 expression in breast cancer can be assumed. It is possible that fulvestrant increased the expression of p44^TMEM26^ and p40^TMEM26^ in MCF-7 and T47D cells, which both highly express RNF115 [35], by downregulating the expression of RNF115. RNF115 is also a target of the PI3K/AKT pathway, which, in breast cancer cells, keeps the expression of this protein high by preventing its proteosomal degradation [30]. However, if the PI3K/AKT pathway activator insulin would act on TMEM26 protein expression through RNF115, insulin would be expected to reduce and not, as observed, to upregulate the levels of p44^TMEM26^ and p40^TMEM26^. Glycogen synthase kinase-3β (GSK-3β) is another regulator of protein stability. By labeling proteins, such as β-catenin or p21, by phosphorylation, GSK-3β triggers their degradation in proteasomes [32, 37]. In MCF-7, inhibition of GSK-3β inhibition can mimic the effect of the proteasome inhibitor MG-132 on proliferation [38]. Both, ERα inhibition by fulvestrant and activation of the PI3K/AKT pathway have been shown to result in the phosphorylation of the GSK-3β at Ser-9 which leads to the inactivation of this enzyme [31, 32]. Hence, both fulvestrant and insulin could potentially block GSK-3β activity in MCF-7 cells and thereby reduce proteosome-dependent degradation of proteins. Future research will reveal the mechanism(s) underlying the fulvestrant- and insulin-mediated changes in TMEM26 protein expression pattern.

As mentioned above, we observed a disconnect between TMEM26 RNA and protein expression supporting the notion that TMEM26 expression is primarily controlled on the protein level. Known examples of other proteins mainly regulated on the protein level are TP53 (tumor suppressor p53) whose activity is regulated by the E3 ubiquitin ligase MDM2 [39] and HIF1α (hypoxia-inducible factor 1α), which in its hydroxylated form is recognized by a pVHL (von Hippel-Lindau)-containing ubiquitin-ligase complex [40]. Nevertheless, the TMEM26-specific siRNA siTM had a strong effect on TMEM26 protein expression in MCF-7 cells and even affected TMEM26 protein levels in MCF-7/FulvR cells, whose TMEM26 RNA level is ~15-fold lower than that in MCF-7 cells. However, not all TMEM26 proteins showed reduced levels in response to siTM. In MCF-7 cells, only the level of p53^TMEM26^ was affected, in MCF-7/FulvR cells additionally the level of p40^TMEM26^. Interestingly, the level of p53^TMEM26^ in the nuclear fraction was never affected, neither by siTM, nor by fulvestrant, tunicamycin or insulin. Since N-glycosylase was able to deglycosylate p53^TMEM26^ in the nuclear fraction to generate p40^TMEM26^, it should be the same protein as that found the plasma membrane and cytosolic fractions. One possible explanation is that p53^TMEM26^ is very stably integrated in the environment from which the nuclear fraction was prepared. The fact that cells needed to be exposed to tunicamycin as long as five days in order to remove substantial amounts of p53^TMEM26^ from the plasma membrane as compared to one day as required for complete withdrawal of integrin β1 from the membrane supports the notion that membrane-bound p53^TMEM26^ is a stable protein.

Though TMEM26 protein expression seems likely to be primarily regulated on the post-transcriptional level, our data also suggest that changes in the TMEM26 RNA level can have significant consequences for TMEM26 protein expression. This is shown with MCF-7 and MCF-7/FulvR cells, whose protein expression is heavily changed after treatment with TMEM26-specific siRNA siTM. Interestingly, the TMEM26 protein expression pattern was the same in fulvestrant-treated MCF-7 cells and fulvestrant-resistant MCF-7/FulvR cells, which both show strongly reduced TMEM26 RNA expression. This suggests that the low TMEM26 RNA expression in MCF-7/FulvR cells is responsible for the altered TMEM26 protein expression in these cells. In T47D cells, fulvestrant resistance was not accompanied by lower TMEM26 RNA expression. Here, the loss of ERα expression may have caused the changes in TMEM26 protein expression pattern. Strangely, siTM increased rather than decreased the cytosolic level of p40^TMEM26^ in MCF-7 cells. Since siTM reduced the expression of ERα in these cells, the decline in ERα expression may have been the reason for this effect. In support of this notion, in MCF-7/FulvR cells, where siTM did not affect ERα expression, the level of p40^TMEM26^ was downregulated.

We show evidence that downregulation of p53^TMEM26^ expression by siTM increases the expression of integrin β1. We further demonstrate that knock-down of TMEM26 and integrin β1 have opposite effects on spheroid formation by fulvestrant-resistant and/or fulvestrant-sensitive MCF-7 and T47D cells and on 3D cell aggregation by ERα-negative SKBR3 cells. This implies that the inhibition of integrin β1 expression by TMEM26 has also consequences for integrin β1 function. Integrin β1 is critically involved in breast development and breast cancer progression. For instance, integrin β1 regulates duct formation and EGF-dependent proliferation in normal breast [41]. In breast cancer, it promotes metastasis formation and drug resistance, including anti-estrogen resistance and is highly expressed in cancer stem cells isolated from ERα-positive breast cancer [9, 42–44]. Hence, the reduced level of p53^TMEM26^ in fulvestrant-resistant cell lines may have contributed to fulvestrant resistance by causing the upregulation of integrin β1 expression. Also, the trend in the group of aromatase inhibitor-treated patients showing that lower TMEM26-specific immunoreactivity may be associated with a more unfavorable outcome may be explained by an increased expression of integrin β1. Hence, by inhibiting integrin β1, TMEM26 may be a potential tumor suppressor. On the other hand, high TMEM26-specific immunoreactivity correlated significantly with a unfavorable outcome of patients diagnosed with ERα-negative (triple-negative) breast cancer. This suggests that, besides inhibiting integrin β1, TMEM26 has additional functions. It is possible that the major functions of TMEM26 are different in ERα-positive and -negative breast cancers.

In summary, our data suggest that, in breast cancer cells, TMEM26 is an ERα-regulated N-glycosylated protein whose function at the cell surface is to control integrin β1 function. By keeping integrin β1 levels low, TMEM26 may suppress the development of anti-estrogen resistance, a notion that is supported by the observation that fulvestrant strongly alters TMEM26 protein expression and by the finding that, in tendency, low TMEM26-specific immunoreactivity correlates with unfavorable outcome of aromatase-treated patients. On the other hand, TMEM26 is also expressed in ERα-negative breast cancer, where a high TMEM26-specific immunoreactivity is significantly associated with unfavorable survival, suggesting different functions of TMEM26 in ERα-positive and -negative breast cancers.

## MATERIALS AND METHODS

### Cell culture

MCF-7, BT474, T47D, BT20 and MDA-MB-231 breast cancer cells were authenticated by SNP analysis (LGC standards, Wesel, Germany or Genolytic, Leipzig, Germany). SKBR3 cells were purchased from ATCC (American Type Culture Collection). Cells were maintained in RPMI medium supplemented with 10% fetal calf serum (FCS, Pan Biotech) in the absence of antibiotics. Fulvestrant-resistant T47D cells, T47D/182^R^-1 and T47D/182^R^-2, were generated as previously described [45]. A fulvestrant-resistant MCF-7 subline (MCF-7/FulvR) was established in our laboratory (Halle) by growing MCF-7 cells long-term in 100 nM fulvestrant (LKT, Laboratories). All cell lines were kept in the same batch of FCS. For treatment with insulin, recombinant insulin (Sigma-Aldrich) was added to cells at a final concentration of 8 μg/ml (~90 μIU/ml).

### Study population

Clinico-pathological data and breast cancer samples from 212 patients who had been treated at the Department of Gynecology of the Otto von Guericke University Magdeburg from 1999-2006 for primary invasive breast cancer were used for a retrospective analysis. These patients represent a subset of a recently published cohort [46]. Selection was done based on the availability of sufficient, archived paraffin-embedded cancer tissue. This study was approved by the Research and Ethical Committee of Otto-von-Guericke University, Magdeburg, Germany. The detailed patient characteristics are summarized in Table 1.

### Immunohistochemistry and -cytochemistry

Paraffin-embedded tissue was sectioned in 3 μm slices. After removal of the paraffin and antigen retrieval in citrate buffer (pH 6) at 125°C for 30 sec, slices were incubated overnight with anti-TMEM26 antibody (Sigma-Aldrich, 1:100) at 4°C. Detection was performed in an automated slide staining instrument (Ventana) by using the iView DAB staining kit (Ventana). The slices were counterstained by hematoxylin and embedded in mounting medium. Staining was scored for area of positive carcinoma cells (area score: 0 = 0%, 1 = 1-9%, 2 = 10-50%, 3 = 51-80%, 4 = 81-100%) and staining intensity (0 = no, 1 = low, 2 = intermediate, 3 = strong staining). These two scores were combined in an IHC score by multiplication and a score of 8 or higher was considered as “high TMEM26” IHC score.

For immunocytochemical staining of TMEM26, cells were grown on Superfrost slides (Menzel) and fixed by using formaldehyde. Anti-TMEM26 reactivity was visualized by using a biotinylated secondary antibody/streptavidin horse peroxidase conjugate-based assay (Zytomed, HRP060) and an AEC substrate kit (Zytomed) by following the manufacturer's instructions.

### RNA interference, growth and spheroid formation assays

RNA interference, cell growth and spheroid formation assays were performed as described previously [10]. For RNA interference the following siRNAs were used: TMEM26-specific siRNA siTM (5′-UCA GCG UCU UCA UAC AAG A-3′), integrin β1-specific siRNA siIB1 (5′-AAG ACU GUG AUG CCU UAC A −3′), SHH-specific siRNA siSHH (5′-CCA GAC UGA GUU AUA AUA A −3′) or control, firefly luciferase-specific siRNA, siL (5′-CUU ACG CUG AGU ACU UCG A-3′). For cell growth analysis, siRNA-transfected cells were incubated for two days, trypsinized, counted and seeded in a 10 cm petri dish at a density of 3×10^4^. After incubation for five days in the absence or presence of 100 nM fulvestrant, the sizes of individual colonies were measured. For spheroid formation assays, transfected cells were seeded onto a layer of 2% Seakem GTG agarose (dissolved in PBS) at a density of 5×10^3^ cells/well (96 well-plate). Cell aggregations were monitored daily for a total of three days. The area occupied by the developing spheroid was used as a measure of the spheroid size as described previously [47]. For analyzing siRNA effects on spheroid formation, cells were transfected with the siRNA and incubated for two days in adherent cultures before the spheroid formation assay was set up. We have previously shown that this procedure allows the siRNA to be effective also after cells were transferred to 3D culturing conditions [47]. Colony and spheroid size measurements were performed by using an AxioCam MRc 5 camera and the AxioVision R 4.5 software (Zeiss).

### Protein deglycosylation

Two μl of MCF-7 protein extract (7.5 μg protein) were mixed with 3 μl 16x deglycosylation buffer (400 mM sodium phosphate, pH 7.4), 0.5 μl protease inhibitor (Sigma-Aldrich) and 9 μl water. The deglycosylation reaction was started by adding either 0.5 μl each of N-glycosidase (PNGase F, New England Laboratories), O-glycosidase (Roche Applied Sciences) and sialidase (Roche Applied Sciences) or by adding 1.5 μl PNGase F alone. For mock-treatment, 1.5 μl water was added to the reaction mix instead of the enzymes. The mixtures were incubated at 37°C for 24 hours and analyzed by Western blot analysis for TMEM26 protein expression. To inhibit N-glycosylation, MCF-7 cells were treated with tunicamycin (Merck Chemicals) at a final concentration of 5 μg/ml for up to five days.

### Measurement of TMEM26 RNA expression

To determine TMEM26 mRNA levels, quantitative RT-PCR was carried out. RNA isolation, cDNA synthesis and quantitative (Q) PCR were performed as described [48]. The RNA isolation kit was purchased from Roche and the dNTP mix was from Qiagen. Briefly, one μg total RNA was used for each cDNA synthesis by using Superscript II (Invitrogen). The PCR-reaction was monitored in a Bio-Rad iCycler after ABsolute QPCR SYBR Green Fluorescein Mix (Thermo Fisher Scientific Biosciences) had been added to the PCR reaction mix. Runs were analyzed by the iQ5 Optical System software version 2.1. The relative RNA level of TMEM26 were calculated by the comparative *Ct* (2^−ΔΔ*Ct*^) method by using GAPDH and HPRT as reference genes for normalization. The primers used for Q-PCR are as follows. TMEM26 (forward: 5′- GAGGGTTGCATCAGCTCCA-3′, reverse: 5′-CGACTCCCGTCACTCAACAAG-3′), GAPDH (forward: 5′- GAAGGTGAAGGTCGGAGT-3′, reverse: 5′- GAAGATGGTGATGGGATTTC-3′), HPRT (forward: 5′- GGACAGGACTGAACGTCTTGC-3′, reverse: 5′- TGAGCACACAGAGGGCTACAA-3′).

### Western blot analysis

Protein extractions and Western blot analysis were carried out as described [48]. Briefly, adherent cells were scraped off, pelleted and resuspended in 400 μl buffer A (10 mM HEPES (pH 7.9), 10 mM KCl, 0.1 mM EDTA, 0.1 mM EGTA). After passing the cell lysate through a 20-gauge needle, nuclear, cytosolic and membraneous protein extracts were prepared by stepwise centrifugation at 3000, 6500 and 13000 rpm in a microfuge as described [49]. Pellets for nuclear protein extraction were treated with buffer C (20 mM HEPES (pH 7.9), 400 mM NaCl, 1 mM EDTA, 1 mM EGTA, 1 mM DTT), pellets for membraneous protein extraction with buffer D (5 mM HEPES (pH 7.9), 0.5 mM K-EDTA (pH 7.2), 1 mM DTT). Protein extracts were separated on a 10% SDS-polyacrylamide gel and transferred to a PVDF membrane (Millipore). The membrane was blocked in 2% skim milk (Applichem), which was dissolved in washing buffer (10 mM Tris/HCl (pH 7.5), 100 mM NaCl, 1 mM EDTA). Incubations of the membrane with the primary and secondary antibodies were done in washing buffer containing 0.2% skim milk. Bands were visualized by chemoluminescence using ECLPlus and Hyperfilm ECL (GE Healthcare). For the detection of the TMEM26 protein, the blot was incubated with the rabbit polyclonal anti-TMEM26 antibody (Sigma Life Sciences) HPA014350 (http://www.proteinatlas.org/) that recognizes the very C-terminus of the protein (Supplementary Figure S1), at a final dilution of 1:500. To assess the specificity of the anti-TMEM26 antibody/protein interaction, 5μl anti-TMEM26 antibody solution (0.1mg/ml) was diluted in 4.5ml milk-free washing buffer and either mixed with 1μl PrEST antigen TMEM26 (3.8 μg/ml, Sigma Life Sciences), the peptide that was used to generate the anti-TMEM26 antibody, or with 1 μl peptide buffer (1M urea dissolved in PBS, pH 7.4). After o/n incubation at 4°C, 0.5 ml of 2% skim milk solution was added to each, the antibody/antigen (TMEM26 antigen) and antibody/buffer (mock) solution. The preincubated antibody solutions were then added to separate Western blots containing the same separated protein extracts. For control reasons, the same experiment was repeated with the anti-Elf-1 antibody: (1:200, Santa Cruz, HC-20.

Other primary antibodies used were as follows. Rabbit polyclonal antibodies: anti-P(S473)-AKT (1:2000, D9E, Cell Signaling), anti-Elf-1 (1:2000, Santa Cruz, C-20), anti-P(Thr202, Tyr204)-ERK1/2, anti-ERK1/2 (both 1:2000, Cell Signaling), anti-E1f-1 antibody: (1:2000, Santa Cruz, HC-20), anti-Her2 (1:1000, Cell Signaling, 29D8), anti-IGF1Rβ (1:2000, Cell Signaling) and anti-SHH (Santa Cruz, H-160); rabbit monoclonal antibody: anti-integrin β1 (1:2000, Abcam, EPR1040Y); mouse monoclonal antibodies: anti-(pan)AKT (1:1000, Cell Signaling, 40D4) and anti-E-cadherin (1:5000, BD Transduction Lab.). The secondary antibody conjugate (anti-rabbit/anti-mouse horse radish peroxidase, 1:2000) was from Cell Signaling. Since antibodies against house-keeping proteins, such as β-actin, are not reliable for checking equal protein loading [50], we instead either stained the gel by Coomassie Blue (Blue G, Serva) or visualized proteins transferred to the membrane by Fast Green.

### Statistical analyses

Statistical calculations were performed with SPSS (version 19, IBM). Possible associations of the TMEM26 IHC score with clinico-pathological factors were analyzed by using the χ^2^ test. Recurrence free survival (RFS) and overall survival (OS, breast cancer-specific death) were determined by Kaplan Meier analysis. For statistical analysis the Log rank test was applied. Hazard ratios were calculated by Cox regression analyses. Data obtained from colony growth assays were analyzed by Wilcoxon matched pair test. A p-value of p<0.05 was considered to be statistically significant.

## SUPPLEMENTARY FIGURES AND TABLES


