# Supplementary material for: Expression of transmembrane protein 26 (TMEM26) in breast cancer and its association with drug response
Source: Oncotarget. 2016 May 20;7(25):38408–26. doi: 10.18632/oncotarget.9493 (PMC5122400; doi:10.18632/oncotarget.9493)
Supplement: Supplementary file 1 [file oncotarget-07-38408-s001.pdf]

SUPPLEMENTARY FIGURES

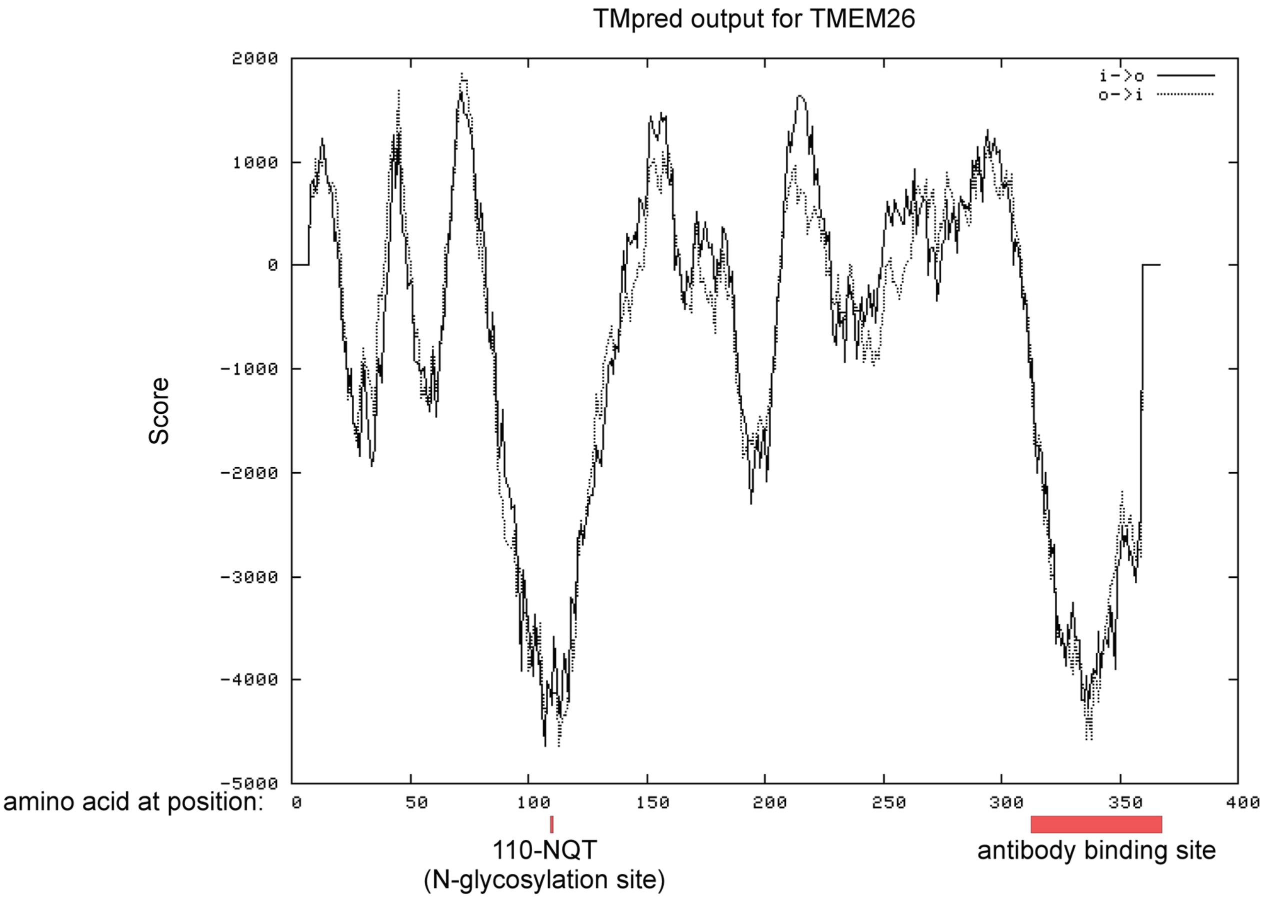

Supplementary Figure S1: Prediction of TMEM26 transmembrane regions and orientation as calculated by TMpred (http://www.ch.embnet.org/software/TMPRED_form.html). Sequences with scores above 500 are considered to form potential transmembrane helices. Six to seven transmembrane helices are predicted by TMpred. The positions of the N-glycosylation site at N-110 and the antibody binding site between R-312 and T-367 are indicated in red.

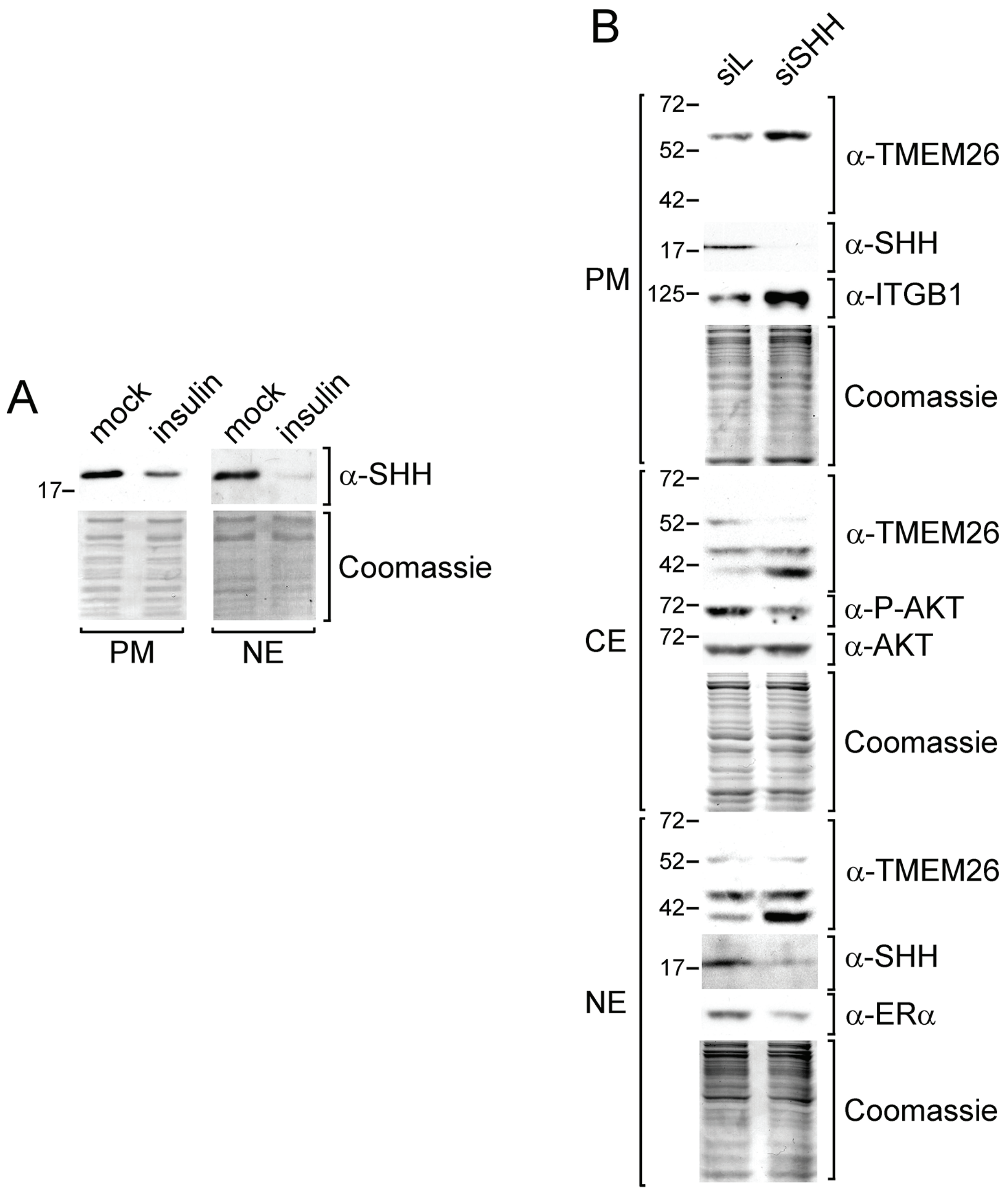

Supplementary Figure S2: A. Insulin induces a decline in SHH (sonic hedgehog) protein expression in MCF-7 cells as determined by Western blot analysis. **B.** Effect of down-regulation of SHH by siRNA (siSHH) on the expression of TMEM26 protein and other proteins in MCF-7 cells. To check for equal protein loading, the proteins that remained in the gel after protein transfer were stained by Coomassie Blue. PM = plasma membrane extract, CE = cytosolic extract, NE = nuclear extract, siL = control siRNA, ITGB1 = integrin β1.

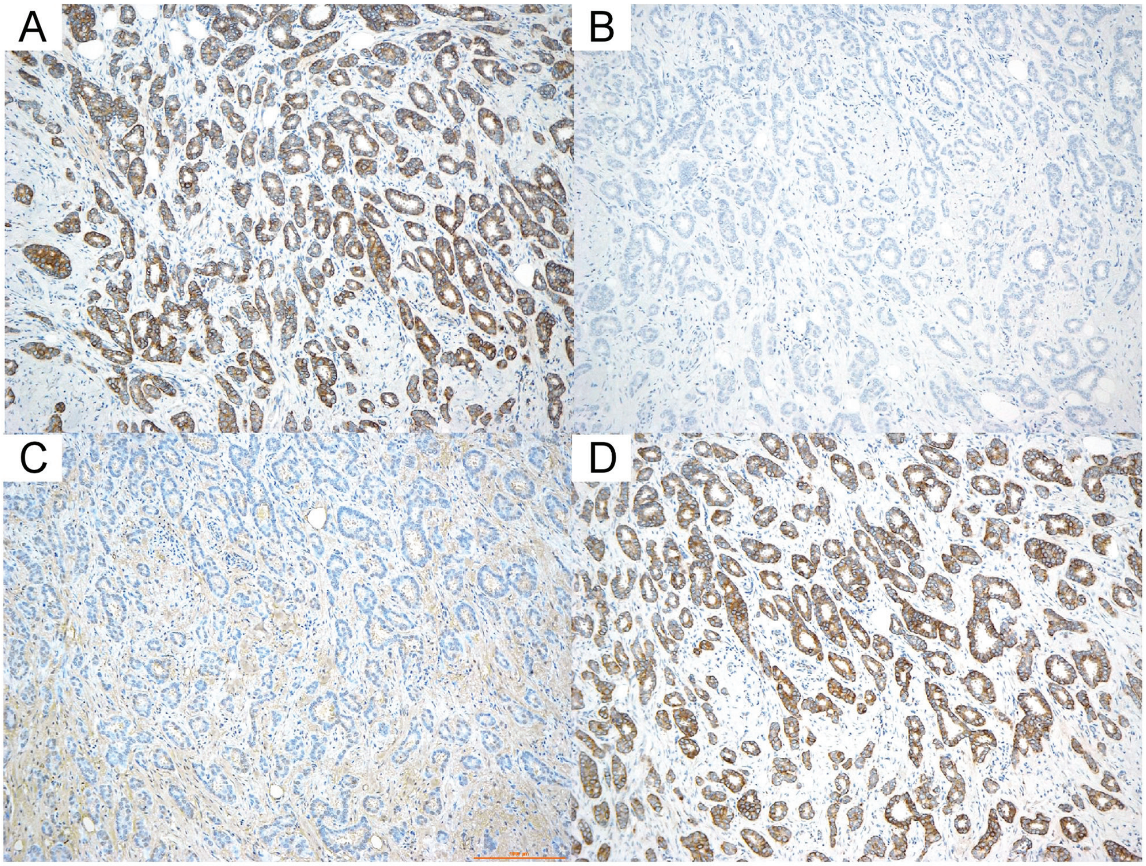

Supplementary Figure S3: Specificity of the anti-TMEM26 antibody reaction in immunohistochemistry. Consecutive sections of a breast cancer specimen were either incubated with **A.** or without **B.** anti-TMEM26 antibody or exposed to TMEM26 antigen-blocked **C.** or mock (buffer)-treated **D.** anti-TMEM26 antibody (antibody to antigen ratio ~ 1:100).

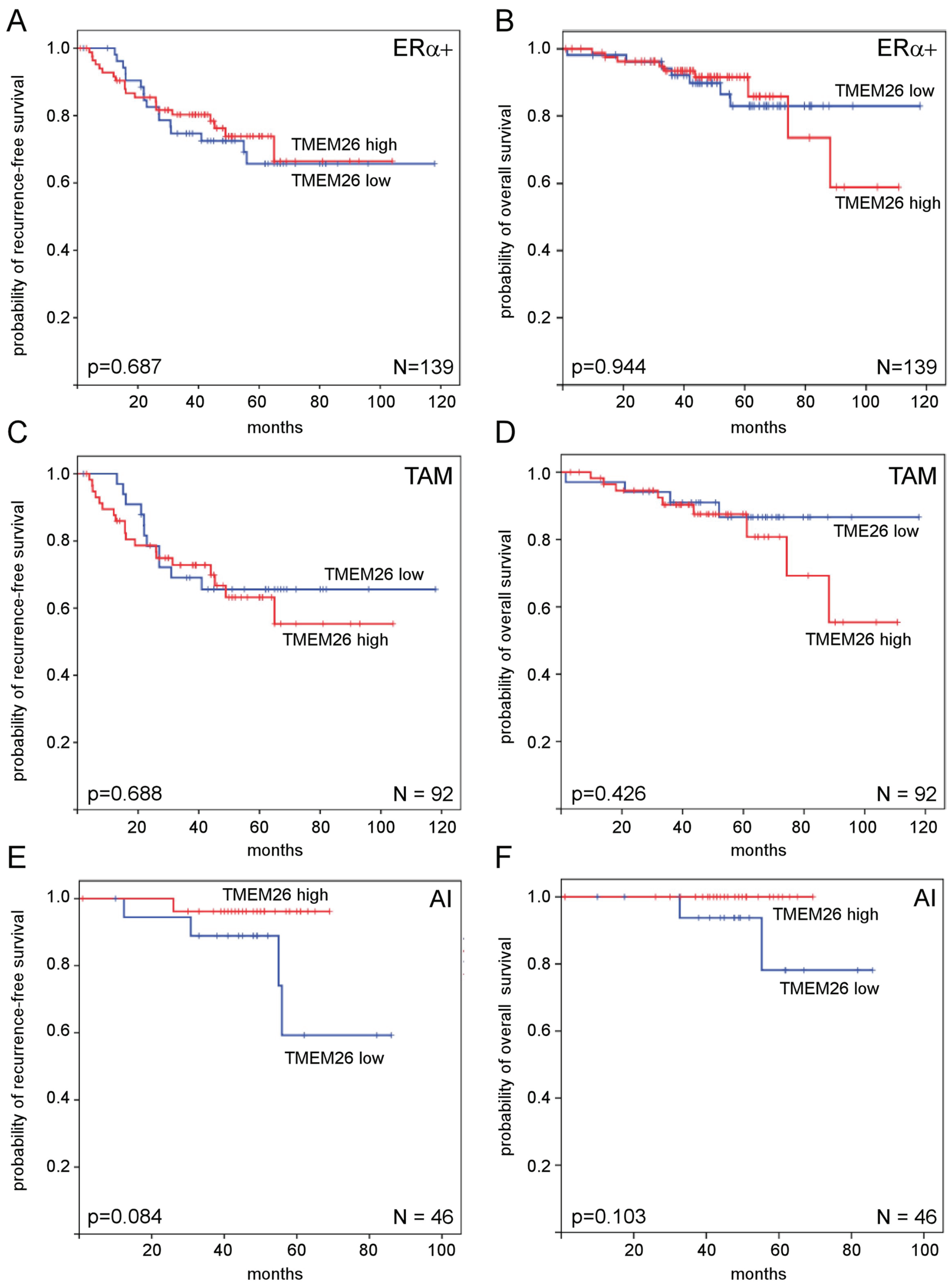

Supplementary Figure S4: Low anti-TMEM26 immunoreactivity is tendentially associated with the response to aromatase inhibitors. The impact of high and low tumoral anti-TMEM26 reactivities on the probabilities of recurrence-free survival **A, C, E.** and overall survival **B, D, F.** were determined for patients who were diagnosed with ERα-positive tumors by using Kaplan-Meier analysis. (C-F) Subgroup analyses of patients treated either with tamoxifen (TAM) **C, D.** or with an aromatase inhibitor (AI) **E, F.**. Log-rank test was applied to determine p-values.

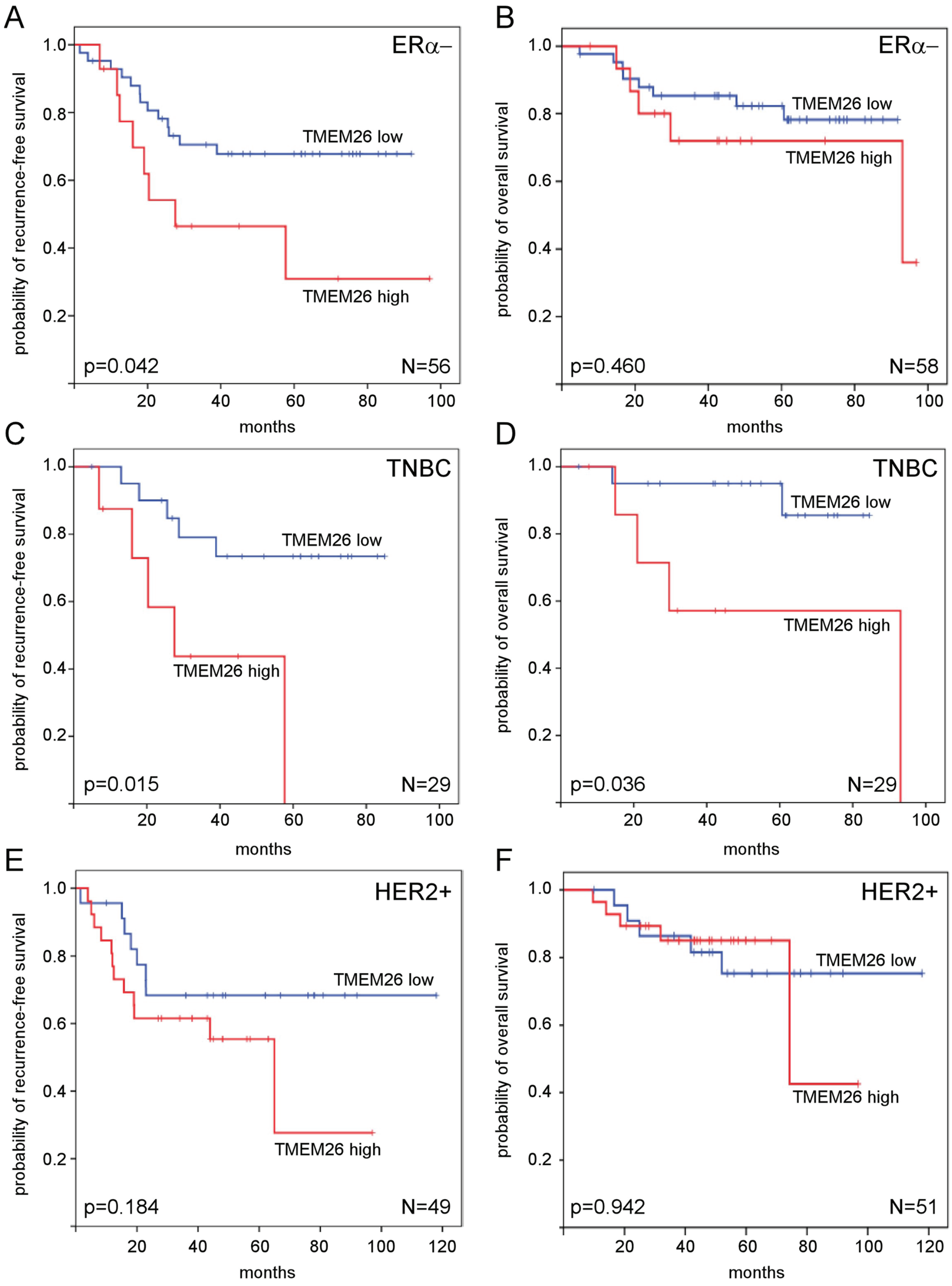

Supplementary Figure S5: High anti-TMEM26 immunoreactivity is significantly associated with unfavorable outcome of patients diagnosed with triple-negative tumors. The impact of high and low tumoral anti-TMEM26 reactivities on the probabilities of recurrence-free survival **A, C, E.** and overall survival **B, D, F.** were determined for patients who were diagnosed with ERα-negative tumors **A, B.**, triple-negative (TNBC) **C, D.** and Her2-positive breast cancers **E, F.** by using Kaplan-Meier analysis. **C-F.**. Log-rank test was applied to determine p-values.

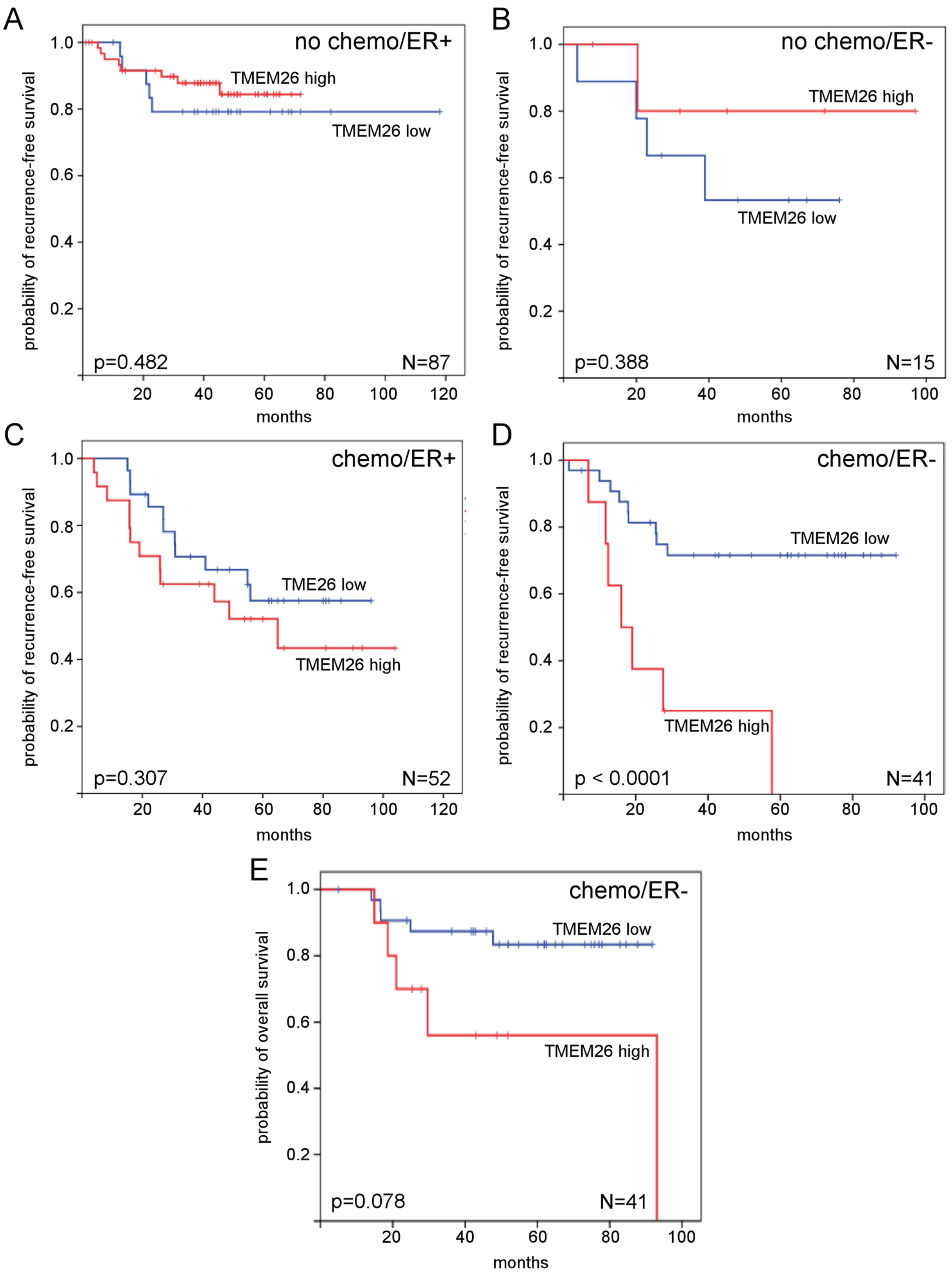

Supplementary Figure S6: High anti-TMEM26 immunoreactivity is significantly associated with unfavorable outcome of patients who developed ERα-negative tumors and received chemotherapy. The impact of high and low tumoral anti-TMEM26 reactivities on the probabilities of recurrence-free survival **A-D.** and overall survival **E.** were determined for patients who received chemotherapy **C-E.** or did not **A, B.** by using Kaplan-Meier analysis. Analyses were done separately for patients who were diagnosed with ERα-positive tumors **A, C.** and ERα-negative tumors **B, D, E.**. Log-rank test was applied to determine p-values.
